# Feasibility and Acceptability of a Digital Intervention to Support Shared Decision-making in Children’s and Young People’s Mental Health: Mixed Methods Pilot Randomized Controlled Trial

**DOI:** 10.2196/25235

**Published:** 2021-03-02

**Authors:** Shaun Liverpool, Julian Edbrooke-Childs

**Affiliations:** 1 Evidence-Based Practice Unit Anna Freud National Centre for Children and Families University College London London United Kingdom; 2 Faculty of Health, Social Care & Medicine Edge Hill University Ormskirk United Kingdom

**Keywords:** mental health, pilot projects, child, adolescent, parents, shared decision making

## Abstract

**Background:**

Interventions to involve parents in decisions regarding children’s and young people’s mental health are associated with positive outcomes. However, appropriately planning effectiveness studies is critical to ensure that meaningful evidence is collected. It is important to conduct pilot studies to evaluate the feasibility and acceptability of the intervention itself and the feasibility of the protocol to test effectiveness.

**Objective:**

This paper reports the findings from a feasibility and acceptability study of Power Up for Parents, an intervention to promote shared decision-making (SDM) and support parents and caregivers making decisions regarding children’s and young people’s mental health.

**Methods:**

A mixed method study design was adopted. In stage 1, health care professionals and parents provided feedback on acceptability, usefulness, and suggestions for further development. Stage 2 was a multicenter, 3-arm, individual, and cluster randomized controlled pilot feasibility trial with parents accessing services related to children’s and young people’s mental health. Outcome measures collected data on demographics, participation rates, SDM, satisfaction, and parents’ anxiety. Qualitative data were analyzed using thematic analysis. Google Analytics estimates were used to report engagement with the prototype. Outcomes from both stages were tested against a published set of criteria for proceeding to a randomized controlled trial.

**Results:**

Despite evidence suggesting the acceptability of Power Up for Parents, the findings suggest that recruitment modifications are needed to enhance the feasibility of collecting follow-up data before scaling up to a fully powered randomized controlled trial. On the basis of the Go or No-Go criteria, only 50% (6/12) of the sites successfully recruited participants, and only 38% (16/42) of parents completed follow-up measures. Nonetheless, health care practitioners and parents generally accessed and used the intervention. Themes describing *appearance and functionality, perceived need and general helpfulness, accessibility and appropriateness,* and *a wish list for improvement* emerged, providing valuable information to inform future development and refinement of the intervention.

**Conclusions:**

Owing to the high attrition observed in the trial, proceeding directly to a full randomized controlled trial may not be feasible with this recruitment strategy. Nonetheless, with some minor adjustments and upgrades to the intervention, this pilot study provides a platform for future evaluations of Power Up for Parents.

**Trial Registration:**

International Standard Randomized Controlled Trial Number (ISRCTN) 39238984; http://www.isrctn.com/ISRCTN39238984.

**International Registered Report Identifier (IRRID):**

RR2-10.2196/14571

## Introduction

### Background

Shared decision-making (SDM) is an ethical imperative whereby health care professionals collaborate with service users to derive care and treatment decisions. The process involves an exploration of health care options, service users’ values and preferences, and achieving treatment consensus [1]. There is a wealth of knowledge suggesting that adopting SDM practices is associated with better outcomes across health care settings [2,3]. However, implementing SDM in children’s and young people’s mental health (CYPMH) services remains a challenge. Barriers to implementation include professional, relational, service user or parent, and service-level and context-level factors [4]. Researchers agree that a primary reason could be the unique triad relationship involving multiple decision makers [5,6]. As a result, parents may navigate between feeling excluded from services, advocating or assuming the role of surrogate decision makers depending on the age and capacity of the child or young person [7,8]. Such feelings and roles sometimes result in added stressors for the parents involved [7]; low service engagement [9,10]; and treatment disagreements between parents, health care practitioners, and young people [11].

Parents and carers are recognized by the literature and by the law as key members of the CYPMH decision-making process [12,13], reporting significant benefits to involvement [14]. However, research in general pediatric care highlights parents’ emotional states as a commonly reported barrier to adopting SDM measures [15]. Despite a range of interventions and service delivery models to support parents’ involvement, time, accessibility, and appropriateness of the interventions also appear to influence use and successful implementation [16]. That study also revealed that research exploring available interventions is limited in that it targets specific populations (eg, attention-deficit and hyperactivity disorders or autism spectrum disorders), uses less innovative modalities (eg, face-to-face, paper-based, or static digital tools), or evaluates interventions using nonrandomized study designs (eg, pre or post, qualitative, or pilot trials). In addition, there appears to be a large number of interventions being developed and implemented without being tested for effectiveness or using small or unrepresentative samples [16].

In response to this, an evidence-based, theoretically informed interactive web-based app was designed and developed to support SDM in universal CYPMH services [17]. The core content and aims of the intervention are based on an earlier intervention named Power Up, an intervention designed to promote SDM among young people accessing mental health services [18,19]. This intervention builds on previous versions by using an affective-appraisal approach that takes into account the emotional states of parents and caregivers.

With the growing interest in applying digital technology in CYPMH services, several modes of delivery, including website and text messages, have been adopted [20]. In line with the Technology Acceptance Model (TAM), it is recommended to test the acceptance of new digital interventions to ensure successful implementation [21]. The TAM is an extension of the Theory of Reasoned Action that focuses on behavioral intention and attitude. The theory proposes that the assessment of perceived usefulness and perceived ease of use can determine whether users engage with the new digital intervention. However, researchers and clinicians agree that poorly designed studies to evaluate these interventions can result in false-positive findings and loss of research investments [22,23].

The evidence-based approach to evaluating effectiveness recognizes randomized controlled trials (RCTs) as the *gold standard* for generating the highest level of evidence. RCTs are viewed as the most rigorous when it comes to determining cause-effect relationships between treatment and outcomes [24]. However, to ensure a successful RCT, it is highly recommended that pilot and feasibility studies are first conducted [25,26]. The Medical Research Council’s guidelines highlight that assessing the feasibility allows researchers to examine important components of the research, such as testing the procedures, estimating rates of recruitment and retention of participants, and determining the sample sizes for future trials [27]. Therefore, acknowledging the relevance of pilot and feasibility studies and in keeping with the Medical Research Council’s framework for developing, evaluating, and implementing a complex intervention, this study was considered an important step.

### Aims and Research Questions

This pilot feasibility study focused on obtaining end users’ views of the intervention and exploring justifiable administration procedures to inform a full RCT. The primary aim is to investigate whether it is feasible to conduct a prospective RCT of an evidence- and web-based app (Power Up for Parents [PUfP]) to promote SDM in families accessing CYPMH services. In addition, this study assessed the perceived usefulness and acceptance of the intervention to determine whether end users would engage.

The following research questions (RQs) were addressed:

Quantitative RQs:

- RQ1: What are the eligibility, consenting, adherence, and engagement rates of participants using PUfP?
- RQ2: Are the outcome measures appropriate and acceptable for a prospective RCT?
- RQ3: What are the potential barriers and enablers to conducting a prospective RCT?
- RQ4: Which data collection procedures are appropriate and acceptable?
- RQ5: What is the scope of the pilot data collected from users and nonusers of PUfP?

Qualitative RQs:

- RQ6: Is PUfP acceptable and useful for parents and health care practitioners?
- RQ7: Can the feedback from PUfP users be used to further refine the prototype for prospective RCTs?

## Methods

### Changes to Protocol

During the initial stages of the study, it was discovered that the intervention may be applicable to settings beyond the CYPMH services offered by the United Kingdom’s National Health Service (NHS). Parent experts in patient and public involvement (PPI) sessions confirmed this by expressing that the intervention was something they could use with limited guidance. In addition, typical service users accessing CYPMH support via the NHS were below the age of 18 years. In line with the United Nation’s definition of young people [28], this research interest extended to parents of young people up to the age of 24 years. Therefore, to obtain more feedback and usage data during the feasibility and pilot testing phases, we added a second recruitment strand (community sampling). It also became clear at later stages of the study that recruitment from NHS services was slower than anticipated, and therefore, the second recruitment strand assisted in increasing the study’s sample size. This change also strengthened the study by allowing further exploration of different recruitment strategies to partly address the aims of the feasibility study.

### Study Design

A mixed methods study involving qualitative data collection and feasibility testing was adopted. Interviews and focus group discussions (FGDs) required user testing of the intervention by health care practitioners and parents to obtain feedback on acceptability and usefulness, and suggestions for further development and upgrading of the prototype. The second stage of the study included (1) a multicenter, 3-arm, randomized controlled, pilot feasibility trial with parents accessing NHS CYPMH services to explore efficiency and eliminate possible study contamination and (2) a web-based individually randomized trial with a community sample of parents to inform recruitment strategies for future trials.

### Study Setting

A total of 18 NHS Trusts in England offering CYPMH services were identified as potential study sites. Community samples were recruited on the web through social media advertising. Parents in the community sample accessed the study via a link to the recruitment software Gorilla [29].

### Intervention: PUfP

The development and evidence base for the PUfP prototype are described and outlined in more detail in the study protocol [17]. PUfP is a decision support intervention with 5 key features (ie, decisions, goals, journey, support, and resources). The intervention aims to encourage discussion, allow parents to ask questions during sessions or seek further information between sessions, and allow health care practitioners to tailor the SDM process to accommodate the needs of the parent and child or young person.

### Participants

#### Health Care Practitioners

A contact person (site collaborator) circulated information about the study to all health care practitioners at the CYPMH services. Then, the health care practitioners attended an information session where a brief introduction and further details of the study were provided. Any health care practitioner who identified as being in contact with the families accessing care when making care and treatment decisions was eligible to participate in the study.

#### Parents

On the basis of the eligibility criteria, the health care practitioners identified the eligible participants. Posters and flyers were posted at the participating NHS sites. To obtain a community sample, the study was advertised on the Anna Freud National Centre for Children and Families’ website between June and August 2019 and promoted through social media platforms (ie, Facebook and Twitter). In addition, a blog post was written on the Association of Child and Adolescent Mental Health’s website to further advertise the study [30]. The recruitment process was guided and informed by the PPI activities and the study’s steering committee.

All parents were screened against the eligibility criteria developed before the start of the trial. Parents were included on the following criteria:

1. Over the age of 18 years
2. No known mental health diagnosis
3. Ability to speak and understand English
4. Parent of at least one child or young person attending CYPMH services.

Parents were excluded on the following criteria:

1. Concurrent and/or involvement in other research that was likely to interfere with the intervention
2. Parents or guardians in cases where the child or young person was being treated under a section of the Mental Health Act.

### Procedure and Materials

#### Qualitative Data Collection

Semistructured interviews and FGDs were conducted. The interview guide aimed to capture the perceived usefulness and acceptability of the intervention, including suggestions for content and prototype upgrade. At the end of the interview sessions, participants were debriefed and encouraged to contact researchers with any further concerns or suggestions.

#### Quantitative Data Collection

Study sites were randomly assigned to either the control group or one of the 2 intervention groups. Intervention group 1 (IG1) received the prospective version 1 of PUfP which included *decisions*, *goals*, *journey*, *support,* and *resources* features. Intervention group 2 (IG2) received version 2 of PUfP without the *support* and *resources* features. The control group included participants who were not exposed to either version of the intervention. The cluster randomization for the NHS sample was completed independent of the research team. For the community sample, participant-level randomization was conducted using Gorilla recruitment software. Therefore, any parent accessing any form of CYPMH service (eg, school mental health support or private therapeutic services) coming in contact with the study information had a chance to participate.

Participants met with a researcher at a convenient time and completed a battery of baseline and follow-up questionnaires. These consisted of demographic details (gender, ethnicity, first language, relationship to child, and child’s age), participation rates (completion of consent, pretest and posttest measures, and intervention use), SDM measures (the Control Preferences Scale for Pediatrics [31], the Pediatric Shared Decision-Making Questionnaire (modified) [32], and the Decisional Conflict Scale [DCS] [33]), experience of service (the Experience of Service Questionnaire [ESQ] [34]), usability and acceptance (the Poststudy Usability Questionnaire [35]), and an anxiety measure (the Spielberger State Anxiety Inventory Form for Adults [36]). Further details on the outcome measures are presented in the study protocol [17]. Depending on which group the participants were recruited into (ie, IG1, IG2, or control), they received help to access the app and were given a guided tour. Participants were then encouraged to use the app as much as they needed to. Participants completed follow-up measures at 3 months after or at dropout or discharge (whichever came first).

The health care practitioners completed an adapted version of the Control Preferences Scale to highlight observed changes in the amount of parental involvement in the child’s care and treatment decisions. Clinicians were asked to select 1 of 5 statements on whether “the parent left all mental health care and treatment decisions about the child to the practitioner” or “the parent shared responsibility for care and treatment decisions with the practitioner.”

At the end of the pilot testing phase, participants were encouraged to share their opinions on the study before being debriefed and thanked for their participation. The Standard Protocol Items: Recommendations for Interventional Trials (SPIRIT) diagram [37] as reported in the study protocol illustrates the participants’ pathway through the trial.

### Data Management and Analysis

#### Qualitative Data

All interviews and FGDs were audiorecorded and transcribed. The data were analyzed using thematic analysis [38]. Data were coded using a combination of a priori themes as categories and emergent themes [39]. The first step generated initial codes from open coding, in which units of meanings were derived from line-by-line analysis followed by axial coding (ie, locating linkages between data) to integrate and differentiate among subcategories [39]. A priori themes or categories were important in framing the emergent themes and assisted in reporting the findings. NVivo was used as the qualitative data management software [40]. An independent investigator reviewed 3 random transcripts and generated codes. The codes were compared and discussed to reach a consensus before inclusion.

#### Quantitative Data

Descriptive statistics were calculated for participant characteristics at baseline, and Google Analytics estimates [41] were used to report engagement with the prototype. To address the aims of the feasibility study, the main focus was on descriptive data. However, some exploratory significance testing using means and CIs were conducted on within- and between-group mean differences at the 2 time points (ie, baseline and follow-up) on the SDM measure using the *as-per-protocol* approach. The intraclass correlation was also calculated to prepare information for sample size calculation within a clustered randomized trial. Analyses were conducted using the Statistical Package for Social Sciences software [42]. Outcomes from both study designs were then tested against 8 Go or No-Go criteria (Multimedia Appendix 1). The criteria were informed by the key areas of focus for evaluating a feasibility study [26]. Upon completion of the study, the following decisions were possible:

- Ready to proceed to full RCT
- Ready to proceed with some action to be taken
- Not ready to proceed to full RCT.

### Recording Adverse Events

Adverse events were identified as any untoward medical occurrence in a parent, child or young person, or HCP, which did not necessarily have a causal relationship with the intervention. Any adverse events arising during the study period were assessed for severity, causality, seriousness, and expectedness (ie, relating to the information provided by PUfP).

### Ethical Approvals and Research Governance

The study was ethically reviewed by the London Surrey Research Ethics Committee (REC) and approved by the Health Research Authority (IRAS 236277) for recruitment at the CYPMH services provided by the NHS. Recruitment for the community sample was approved by the University College London REC.

## Results

### Overview

Recruitment was expected to begin in October 2018 and was scheduled to last for 1 year. However, NHS REC approval was received in December 2018; therefore, recruitment from NHS began in January 2019. We approached 18 NHS sites, of which 12 (67%) CYPMH sites expressed interest and were recruited to participate in both stages of the study. Web-based community advertising resulted in 387 unique visitors on the study webpage. The data collection was terminated on October 1, 2019. The results section is structured according to the study’s RQs.

### RQ1: What Are the Eligibility, Consenting, Adherence, and Engagement Rates of Participants in the Trial?

Through consultation with site collaborators and HCPs, the eligibility criteria were considered to be clear and straightforward. However, one site expressed difficulties in recruiting parents due to the high percentage of parents at that site with a mental health diagnosis, which met the exclusion criteria. Consequently, this site withdrew from the study within 3 months of confirming its capacity and capability.

#### Qualitative Study

A total of 40 parents consented to participate in interviews or FGDs (ie, 36 from the NHS and 4 from community recruitment). In total, 36 parents from the NHS were recruited from 58% (7/12) of the participating sites. The remaining 5 sites not recruiting participants included the site that withdrew from the study, 1 site that wished to take part in the quantitative study only, and 3 sites that stated that the parents were too busy to commit to an interview or FGD. Consequently, a total of 24 parents participated (24/40, 60%): 14 parents were interviewed, and 10 participated in FGDs. For the remaining participants who consented but did not attend an FGD or interview, it was not possible to contact them on the email or phone contact provided by the site collaborator or to arrange a convenient time for an interview. The sample included 22 mothers and 2 fathers with a mean age of 44.9 (SD 6.76) years. The majority of the sample (23/24, 96%) was of White or White British ethnicity. The mean age of their children was 13.88 (SD 2.8) years, and the children were experiencing a range of mental health problems. Of the children, 29% (7/24) were boys, 66% (16/24) were girls, and 4% (1/24) identified as other (Multimedia Appendix 2).

A total of 33 HCPs from 8 NHS sites were included in the study. In total, 19 of the 33 participants were interviewed, and 12 participants participated in the FGDs. For the remaining 2 HCPs (6%), it was not possible to arrange a time that was convenient during the recruitment period. HCPs represented a broad range of clinical expertise, worked with children and young people aged from 0 to 25 years in an outpatient capacity and had an average of 7.54 (SD 6.24) years of working experience in CYPMH services (Multimedia Appendix 3).

#### Quantitative Study

**Figure 1:**
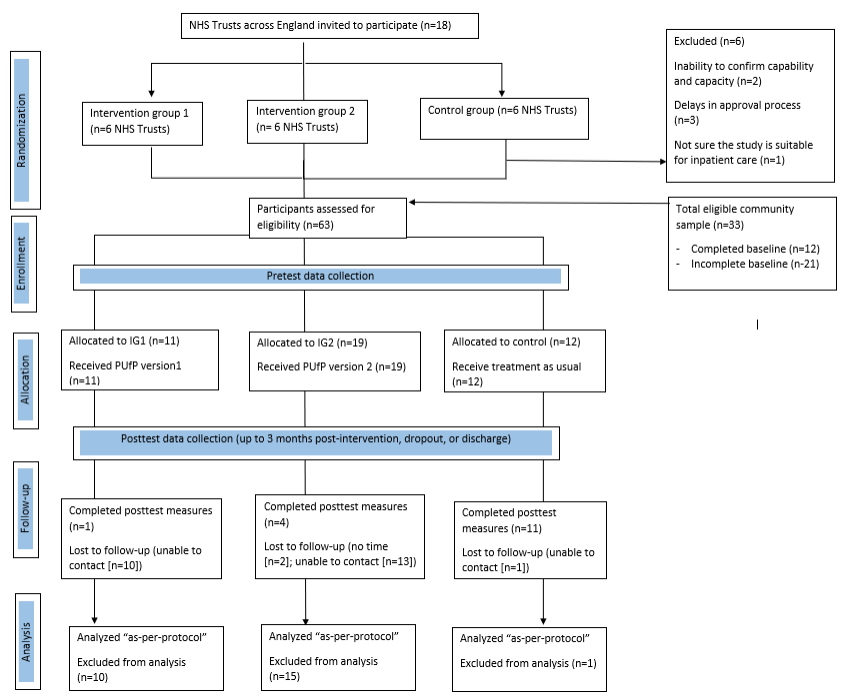
Consolidated Standards of Reporting Trials flowchart of participants in the quantitative study. IG1: intervention group 1; IG2: intervention group 2; NHS: National Health Service; PUfP: Power Up for Parents.

A total of 63 parents met the eligibility criteria and consented to be part of the pilot RCT (ie, 30 from the NHS and 33 from the community sample; Figure 1). There were no significant demographic differences in the parents accessing the trial through community recruitment and those accessing through the NHS (*χ*^2^_8_=8.272; *P*=.41). Of the 63 parents, 42 (67%) parents completed baseline measures (30 from the NHS and 12 from the community sample) and were randomly assigned to control (n=12), IG1 (n=11), or IG2 (n=19). Of the 42 parents, 16 (40%) completed follow-up measures (ie, 12 from the NHS and 4 from the community sample). A total of 2 parents expressed not having time to complete the follow-up measures, and the remaining parents could not be reached. There were no significant differences between the parents who consented and completed baseline measures and those who consented but did not complete baseline measures (*χ*^2^_8_=8.766; *P*=.36). Similarly, there were no significant differences between the parents who completed follow-up measures and those who did not (*χ*^2^_8_=8.015; *P*=.43).

Only 50% (6/12) of the NHS sites were able to recruit parents to stage 2 of the study with an intraclass correlation of 0.042 on the Pediatric Shared Decision-Making Questionnaire (modified). Of the remaining 6 sites, one withdrew from the study and another site reported insufficient clinical staff to assist in identifying potential parents. The other 4 sites entered the study within the last 3 months of recruitment and reported insufficient time to recruit participants for both stages of the study. The randomized sample (n=42) was predominantly White British, English-speaking mothers, with a mean age of 45.98 (SD 6.45) years. The majority of the participants were primary caregivers of teenage girls with a mean age of 14.31 (SD 2.14) years (Multimedia Appendix 4).

#### Engagement With the Intervention

Google Analytics use data from January 7, 2019 and October 1, 2019, were used, as the data coincided with the recruitment of the first participant to the pilot study and the last day of data collection. App use data were made anonymous to comply with the General Data Protection Regulation and research ethical guidelines. Overall, 117 users cumulatively accessed versions 1 and 2 of the app and 72 registered an account. In total, users visited the app 288 times for an average duration of 5 min and 59 s. Less than 33% of the users visited the app and left immediately without viewing any of the features (bounce rate=32.99). An average of 3 active users were recorded for each 28 day-period during the study. The *decisions* feature was accessed 330 times, followed by *journey* 163 times, *goals* 160 times, *resources* 146 times, and *support* 103 times. All parents recruited via the NHS were guided through the setting up of the app, and web-based participants had to download the app before clicking next to indicate completion of baseline measures. Therefore, it was estimated that the majority of the intervention arm participants (n=30) accessed the intervention contributing to these statistics.

### RQ2: Are the Outcome Measures Appropriate and Acceptable for a Prospective RCT?

For parents who completed baseline measures (n=42), the majority (40/42, 95%) had no missing data at baseline. The 2 cases with missing data failed to complete the Pediatric Shared Decision-Making Questionnaire (modified) and the DCS. For parents completing follow-up measures (n=16), all measures were completed by all parents, except the Poststudy System Usability Questionnaire (PSSUQ). Only parents belonging to the intervention groups were required to complete the PSSUQ, and all 5 completed it. At baseline, 53% (16/30) of the NHS cases had completed the HCP observed Control Preference Scale (CPS). At follow-up, 58% (7/12) of the NHS cases had completed the HCP observed CPS. The HCP observed CPS was required only from parents recruited via NHS.

The outcome measures provided valuable information on parents’ anxiety levels, decision-making preferences, and experiences of SDM. Data from the outcome measures were summarized and descriptively presented (Multimedia Appendix 5). Overall, the majority of parents (n=26) who participated in the study preferred to be involved in SDM. However, clinicians reported that, based on observations, parents either left the final decision to the HCP after sharing their views (n=6), got involved in SDM (n=5), or preferred to make the final decision themselves (n=4). The average Pediatric Shared Decision-Making Questionnaire (modified) score reported at baseline was 26.54, which increased to an average of 28.8 at the end of the study (higher values indicate greater levels of SDM). The average DCS increased from 35.44 to 38.18 during the study (higher values indicate greater levels of decisional conflict). In addition, the average overall satisfaction with care score increased from 20.62 to 26.18 by the end of the study (higher values indicate greater experience of service). However, the SDM construct of the ESQ highlighted that many parents did not experience SDM at baseline (32/42, 76%) and again at follow-up. The average overall anxiety scores for the sample showed scores that were above the cut-off (38) at both time points, indicating that the parents in the sample were moderate to highly anxious. The PSSUQ had a mean score ranging from 3 to 3.4 overall and on all the subscales (lower values indicate better performance and satisfaction).

### RQ3: What is the Scope of the Pilot Data Collected From Users and Nonusers of PUfP?

As the Pediatric Shared Decision-Making Questionnaire scores for the overall sample shifted in a positive direction by the end of the study, this measure was investigated further to gain insight into the SDM outcome. The CIs around the estimated differences in mean scores were too wide to indicate any potential significant differences between the groups [43]. However, based on observed data, at baseline, there was a small observed difference between the control (mean 28.12, SD 9.17) and intervention groups (mean 25.86, SD 11.46) in the Pediatric Shared Decision-Making Questionnaire (2.26 points, 95% CI −5.31 to 9.92). At the end of the intervention period, a small difference was observed between the control (mean 29.36, SD 3.12) and intervention groups (mean 27.6, SD 11.89; 1.76 points, 95% CI −10.75 to 14.28). On the basis of the observations, both the control and intervention groups may have increased the behaviors of SDM over time.

For participants completing both baseline and follow-up, it was observed that the control group at baseline (mean 28.91, SD 29.36) showed very little change at follow-up (mean 29.36, SD 10.36) on the Pediatric Shared Decision-Making Questionnaire scores (−0.45 points, 95% CI −4.75 to 3.84). The intervention group showed a small difference from baseline (mean 22.2, SD 10.62) to follow-up (mean 27.6, SD 11.89; −5.4 points, 95% CI −26.56 to 15.76). Again, CIs around the estimated differences in mean scores were too wide to indicate any potential significant differences over time. These preliminary findings suggest that if the change over time was ignored, parents in the control and intervention groups may have had similar scores on the Pediatric Shared Decision-Making Questionnaire [43].

### RQ4: What Are the Potential Barriers and Enablers to Conducting a Prospective RCT?

Potential barriers observed or reported by site collaborators included insufficient time for recruitment and site setup, as indicated by the challenge sites faced to recruit participants within the final 3 months of the study. Second, including a criterion that excluded parents with a mental health diagnosis decreased the number of potential participants. This was confirmed by the withdrawal of 1 site that expressed challenges with recruitment, as most parents reported having a diagnosis.

Although this pilot feasibility study highlighted potential barriers that can affect recruitment in a full RCT, the study highlighted no reports of adverse effects in either stage of the study. It was also possible to recruit a satisfactory sample (n=31) across 6 NHS CYPMH sites within 9 months and 33 participants within 3 months of community sampling. However, a high attrition was observed among the participants. No other barriers to upgrading to a full RCT were observed or identified. Input from PPI sessions and guidance from the study’s steering committee were highlighted as beneficial to the intervention development and recruitment strategies.

### RQ5: Which Data Collection Procedures Are Appropriate and Acceptable?

For the qualitative study, the majority of parents (12/14, 86%) opted for phone interviews. In addition, those participating in FGDs preferred this to be held at the CYPMH site and attached to an existing meeting, instead of the university. For quantitative data collection, the majority of parents preferred to complete the baseline (30/42, 71%) and follow-up (10/16, 63%) measures on the web.

Similarly, for clinicians participating in the qualitative study, all clinicians opted for phone interviews. Those participating in FGDs preferred this to take place at the CYPMH sites and attached to an existing staff meeting. Although there was no web-based option for HCPs completing the observed Control Preferences Scale, many HCPs requested to have the measure emailed or to receive a reminder email to prompt completion of the measure. In addition, both forms of randomization worked smoothly, with an unpredictable assignment to the comparison groups.

### Qualitative Results

#### RQ6: Is PUfP Acceptable and Useful for Parents and HCPs?

**Figure 2:**
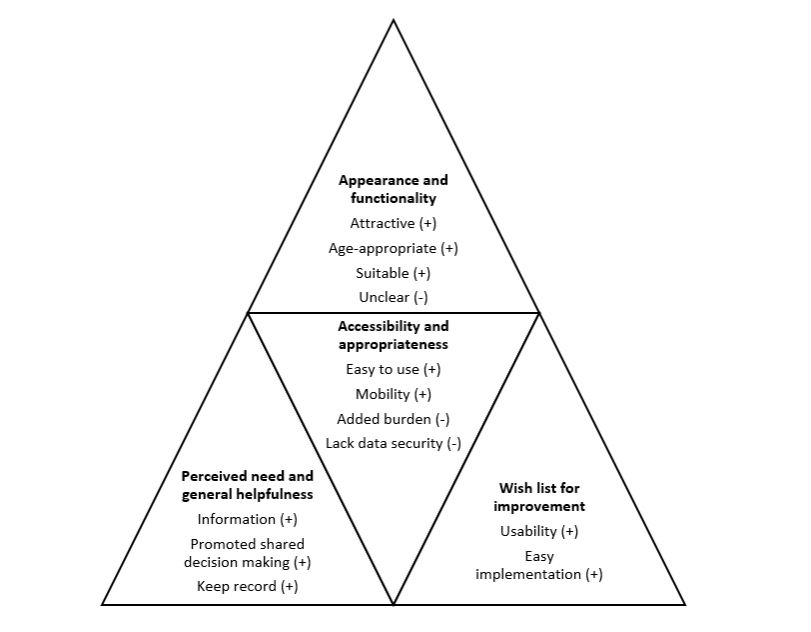
An overview of the themes emerging from qualitative data.

Feedback revealed feasibility categories that represented acceptability, (perceived) usefulness, and scope for improvement. Participants described the *appearance and functionality* of the intervention as essential to the acceptability of PUfP. *Perceived need and general helpfulness of the intervention* and *accessibility and appropriateness* of the intervention emerged as 2 further themes describing the perceived usefulness of PUfP. Figure 2 provides a brief overview of the themes emerging from the qualitative data, highlighting the important influencing factors.

##### Acceptability

###### Theme 1: Appearance and Functionality of the Interface

Parents’ feedback on the intervention was mostly positive, generally expressing satisfaction with the intervention (11/14, 79%). Most parents described the appearance of the intervention as attractive. Parents also appreciated the layout and functionality of the intervention and described it as age-appropriate and suitable for their busy lifestyles. There was a general sentiment that images and graphics were ideal for parents:

I find it much easier on the eye. It gives a soothing vibe kinda thing.

Parent, age 47 years

Yeah, it looks good, colourful.

Parent, age 40 years

It’s not overly childlike. Yeah, I think it looks very user friendly.

Parent, age 53 years

HCPs also expressed satisfaction with the appearance and provided favorable comments on the presentation of the intervention (15/19, 79%). HCPs were also positive in endorsing specific components of PUfP and its suitability for parents. HCPs highlighted that the layout and colors drew attention to the relevant features within the app:

I like the layout, in terms of the different sections. I think that’s really good.

HCP, 2.5 years of experience

It’s nice and clear in terms of the graphics. It tells you what it is, and the tabs are really nice.

HCP, 7 months of experience

Although parents and HCPs were generally satisfied with the intervention, some expressed dislike with some of the features. In addition, not all participants understood all features. Dislikes centered on having a preference for specific colors and wording. Although parents were able to find their way around the app after *clicking around* or browsing the user manual, participants expressed that clarity or further instructions are needed to guide users:

I would say, I don’t like question mark boxes, because I think the text should maybe be in the main box itself, because it’s just another thing to click on.

HCP, 6 years of experience

So it's not altogether clear what that [Support section] does...here I've got a plus and a minus...

Parent, age 51 years

##### Usefulness

###### Theme 2: Perceived Need and General Helpfulness

Parents generally provided positive feedback, highlighting that the intervention was useful (13/14, 93%). The intervention was well received by the parents, and they generally indicated that the intervention was or would be useful for them and may also help with various aspects of accessing CYPMH care. Parents echoed the potential value of the intervention to keep records, promote involvement in SDM, and signpost useful resources:

...and if it worked and it worked well, I’d be using it. It’s really good to have all your appointments in one place as well. And the notes section, things that you think, “Oh, I need to talk to the doctor about that.” Yeah, I think it sounds really good.

Parent, age 39 years

This definitely looks like something I would use.

Parent, age 47 years

Similarly, many HCPs expressed that the intervention was useful and would be relevant to their practice (16/19, 84%). The HCPs provided insight into the potential application and benefits of PUfP, with the majority expressing that it should make it easier to signpost families to useful resources that can support their practice:

It might also be helpful in terms of just understanding CAMHS. That’s often one of the first hurdles that I have to get over with parents and young people, is they don’t really understand our service and they don’t really understand CAMHS. I think that could be quite helpful in this.

HCP, 13 years of experience

I think this can be used with any diagnosis. This is kinda helpful. With any kinda parents, this is helpful.

HCP, 15 years of experience

####### Theme 3: Accessibility and Appropriateness of the Intervention

The concept of an app received mixed views from parents and HCPs, mainly regarding usability. However, participants highlighted positive reasons for using an app and expressed that an *easy to use* and *easily accessible* app may motivate parents to at least try the intervention. Participants generally thought a digital resource provided that *instant* support and because of its dynamic nature may also help the parents by providing feedback and signposting. Participants also expressed appreciation that the intervention had the potential for use *on the go*:

I think even if there were parents with learning difficulties or struggled with using a bit of technology, I think, as long as they obviously had a phone, you know, that they brought with them and we were able to help guide them through it, I think that could still work as well.

HCP, 2.5 years of experience

I think I’d probably use it more on my phone because that’s constantly with me. So, if something happened, like panic attack in McDonalds, like we’ve had before, something like this will be quite handy.

Parent, age 36 years

Although many participants highlighted that the intervention presented limited potential for harm, there were genuine concerns around specific groups of parents, suggesting that the intervention may be an additional burden to parents. Generally, a sense of excluding some users based on their comfort with technology or level of literacy was expressed. Similarly, data security and privacy were also highlighted as concerns. Participants expressed that sensitive data would be entered into the app, and therefore, reassurance of trustworthiness and safety would be needed:

just thinking about culture and ethnicity and language, and whether or not this would be available in different languages, for those that don’t read English, basically.

HCP, 2.5 years of experience

as long as I’m assuming, it’s obviously all secure with the data that you put on there and everything. As long as I was confident that what I was putting on there was all secure.

Parent, age 39 years

Well, I have a few illiterate parents so they may struggle with this.

HCP, 16 years of experience

#### RQ7: Can the Feedback From PUfP Users be Used to Further Refine the Prototype for the Prospective RCT?

The following theme emerged addressing the final RQ.

##### Theme 4: A Wish List for Improvement

Parents and HCPs appreciated that their input could potentially help further develop and improve the PUfP prototype for future research and before implementation. They suggested improvements that could enhance usability and facilitate easy implementation into practice. Feedback was either in line with refining what already existed (eg, attaching the user manual to the home screen) or adding new features that were seen as vital (eg, emergency help) or features that could promote use of the app (eg, options for emotional support such as mindfulness). The overarching theme emerged as *a wish list of improvements* for informing the development and refinement of PUfP:

A section on mindfulness, for themselves...

HCP, 2.5 years of experience

Sometimes a brief video of how to use the app can be useful, or testimony of another parent or carer talking about themselves can be helpful.

HCP, 5 years of experience

I think if there was under resource, if there was things like, “If this happens, do this.” Maybe that would help.

Parent, age 47 years

Maybe having the manual where it is fine, but maybe there could be a smaller, I don’t know, more compact, sorry, more compact version within the app itself to just remind people what each of the particular areas are for.

Parent, age 39 years

#### Overall Feasibility and Acceptability

The findings suggest that although the components of the study work well together, adjustments to the study protocol to improve recruitment are needed to proceed to an appropriately powered prospective RCT. In addition, the intervention appears to be acceptable and usable, with findings further suggesting upgrades and improvements that may benefit future trials. On the basis of the 8 Go or No-Go criteria (Multimedia Appendix 1), this study achieved 15 points out of a possible 16. Although many points were accumulated throughout the study, on further reflection, we acknowledge the importance of an adequate sample size to facilitate a fully powered RCT. This is an important issue; therefore, we were particularly cautious in our interpretation of the points-based system.

## Discussion

### Summary

This study was a preliminary investigation to pilot PUfP, a novel digital evidence- and web-based app to promote SDM among parents of children and young people with mental health difficulties. This study aimed to assess the acceptability of the intervention and examine the feasibility of proceeding to a full RCT. To the best of our knowledge, this is the first RCT to pilot test an interactive parent-targeted digital SDM tool in CYPMH settings. Qualitative data revealed that PUfP may be acceptable and useful for parents and HCPs. The findings also indicate that there is scope for improvement of PUfP with suggestions for refinement and upgrade. A total of 63 parents met the eligibility criteria and consented to participate in the pilot RCT. However, 42 completed baseline measures and only 16 completed follow-up. Although there is some evidence indicating that general administrative procedures such as overall study design and selection of outcome measures are appropriate for a future RCT, the high attrition of participants suggests that some modifications should be applied to increase recruitment target numbers before upgrading to a fully powered RCT.

### Results in Context With Other Research

The 15 points accumulated from the Go- or No-Go criteria indicated the successful completion of the study [44]. The main potential barrier identified for the future trial centered on recruitment. This is not uncommon among researchers recruiting in medical settings [45]. On the one hand, we obtained data from 24 parents and 31 clinicians, which were acceptable for the qualitative study and permitted data saturation [46]. On the other hand, approximately 50% of the participants were lost to follow-up when assessing feasibility measures. Although high attrition is consistent with research in web-based interventions [47], the small sample size was comparable with other studies exploring decision aids in CYPMH settings [48-52]. In addition, our sample size was higher than the recommended sample size for feasibility and pilot studies [53]. Nonetheless, a larger, more appropriate sample will render the research more efficient.

The number of eligible participants identified through social media (n=33) is consistent with other studies reporting social media as beneficial to recruitment rates [47]. In contrast to previous studies indicating challenges in recruiting HCPs [45,52], this study identified a fair sample (n=24) of interested HCPs. A possible explanation could be that the topic resonated with the clinical care agenda or is in an area of special interest to HCPs at CYPMH sites [54]. Use data, however, demonstrated the feasibility of parents accessing and using the intervention. Similar findings have been reported in the original Power Up for young people tested in schools and CYPMH services [18]. Taken together, these findings highlight that future trials of PUfP should clearly define and discriminate adherence to intervention and adherence to study protocol. The relationship between the 2 types of attrition could have implications for how the findings are interpreted [55]. For future trials of PUfP, adherence to PUfP may promote SDM; however, a meaningful sample size is needed to make the necessary comparisons.

In general, parents reported a preference to be involved in SDM. However, HCPs reported that some parents in their care displayed behaviors in line with SDM, left the decision up to HCP, or made the final decision themselves after sharing their views or listening to the HCP’s recommendations. These preliminary findings are in agreement with other academics, suggesting that although SDM is preferred, not everyone engages [56,57], or it may be too challenging to implement [4,15]. This finding also highlights that, within triad relationships, varying levels of *shared* decision-making may exist [6]. This may be, in part, due to the age and capacity of children and young people.

The majority of the qualitative sample (>80%), including parents and HCPs, provided feedback consistent with the acceptance and (perceived) usefulness of the intervention. These findings demonstrate that additional support is generally well received in CYPMH settings, as indicated in other studies [48,49,52]. The 3 emerging themes highlighted the importance of the intervention for end users and may promote use. These themes also fit within previous research on the broader TAM [21]. Qualitative findings further highlighted *a wish list* of features and improvements to PUfP, which may potentially increase acceptability and usefulness. Incorporating the participants’ views would be in line with human and computer interaction approaches to designing technological interventions and reinforces an opportunity to involve end users in the development of interventions. Researchers generally agree that this approach to co-designing improves usability and subsequent outcomes [58].

Investigations of parent responses to the Pediatric Shared Decision-Making Questionnaire using CIs resulted in no significant findings within and between groups. This is not surprising because of the small sample size obtained and the *as-per-protocol* analytic approach chosen [59]. However, parents’ average anxiety levels were mostly above the cut-off for this study’s sample. This is in line with other research suggesting that parents of children with mental health difficulties generally report higher stress levels [7].

### Strengths and Limitations

The primary strength of this study was the ability to obtain data that could be useful for scaling up to a full RCT. Recruitment figures were improved by including web-based community sampling and social media advertising. Future trials could explore these forms of recruitment further, balancing the possible high proportion of incomplete data via the web-based platform versus the slow recruitment process via the NHS sites. Another strength of this trial was the consideration for respondent burden by providing the participants with options for phone or face-to-face interviews and options for completing outcome measures on the web or paper based. Notably, having limited contact details made it difficult to reach some of the parents and resulted in a small number of parents completing follow-up measures.

Although emerging themes suggested acceptability and perceived usefulness, these themes were informed by views taken from a nonrepresentative sample that included the majority of White British, English-speaking mothers of teenage girls. A more representative sample, including fathers, other ethnicities, or underrepresented groups, can provide deeper insights into future research. A multi-site, cluster randomized approach was considered a major strength and did not incur additional intervention costs. However, potential contamination of the control group could be considered if the participants come in contact with the community study recruitment information. This may present some obstacles for the research team if the control group gained access to the intervention. In addition, a web app was chosen over a native mobile app. As a result, parents were not required to download or install it from an app store. Therefore, PUfP did not occupy space on the user’s phone. It functioned as a website that is suitable for mobile devices and is usually cheaper to build, maintain, and update than native mobile apps [60].

Another major strength was the ability to gather use data via Google Analytics. Although the research team attempted to share the intervention only with the intervention arm, it was possible that site collaborators, HCPs, and parents could have shared the link with nonstudy participants. This may have affected the accuracy of the use data, and therefore, caution should be exercised when interpreting these types of data. Future studies may need to collect both use and self-report data to present a more reliable picture.

Finally, a mixed method design was also viewed as a strength at the feasibility phase of the intervention. Outcome measures provided valuable information that is of importance to the full RCT, providing a basis for estimating sample size calculations and selecting appropriate measures. Similarly, it provided estimates of the time required to complete outcome measures and gain access to the intervention. Although obtaining qualitative and quantitative data from the participants may triangulate the findings, this approach may potentially add a burden to parents. However, the mixed method approach can provide a better understanding of efficacy and efficiency and strengthen the findings of future RCTs [61].

### Implications for Clinicians and Policymakers

Although these findings are preliminary, they suggest potential areas of clinical application. First, PUfP was found to be acceptable, as suggested by the parents and HCPs in our sample. The positive feedback surrounding the theme of perceived need and the general usefulness of PUfP highlighted a desire to obtain support if SDM was to be successfully applied in CYPMH. These findings are in line with those of other researchers, suggesting that policy guidelines should be considered to support parents who report feeling uninformed and excluded from services [54]. Notwithstanding the acknowledgment of the *Gillick competency* principle [62], policy guidelines specific to CYPMH could be informative for HCPs working with families of young people who are still considered being *under the care* of their parents. Finally, the findings also highlighted moderate to high levels of anxiety among parents. This may provide HCPs with a knowledge base for the emotional state of the parent population accessing CYPMH care.

### Future Directions

The generalizability of our findings is unclear. However, the findings suggest that PUfP has the potential to be evaluated in future research. First, it is recommended that PUfP be upgraded and refined in line with the suggestions provided by HCPs and parents before being tested further. These suggestions can impact the usefulness and usability of the intervention. For example, incorporating mindfulness techniques or other techniques can provide additional support to parents during difficult moments. Just as important are the suggestions to include a crisis section and features to facilitate optional communication between HCPs, children and young people, and parents or parent-to-parent interactions. These improvements should also be made in collaboration with end users to ensure the suitability of the components.

In terms of the study design, it is recommended that future trials maintain a multicenter randomized controlled study design. However, a 2-arm approach may be sufficient, as growing evidence suggests that parents involved in mental health decisions may benefit from additional support [2]. Therefore, if a 3-arm study design is to be maintained, the existing body of knowledge may benefit from insights into different modes of delivery. In addition, clustered randomization is recommended to control for site-level activities that can impact family involvement in SDM [14]. However, if community recruitment is also used, comparisons can be made between samples to strengthen the findings or considerations can be made to stratify the community sample into existing clusters.

Another recommendation is to revise the eligibility criteria to allow parents with an existing mental health diagnosis to be included in future trials. These parents may actually benefit from the additional support, and therefore, future trials can control for and benefit from these statistical comparisons. It is also recommended that the future trial adopts an *intent-to-treat* analytic approach to draw accurate (unbiased) conclusions regarding the effectiveness of the intervention [59]. This approach will also be beneficial in light of the retention rates observed in this trial.

The study also benefited from the input of enthusiastic parent partners who contributed to the intervention design and study recruitment strategies. Future studies could use this PPI approach, as it possibly contributed to the smooth running of this feasibility study. Future trials can also explore extending an invitation and training to parent experts so that they can be part of the research process as interviewers or participate in the identification and recruitment process at CYPMH services. Finally, it may also be possible to estimate NHS provider costs for usual care and other interventions, in addition to the parent-reported costs to access services. Taken together, these costs can be explored to fully capture any savings to be estimated if the future trial incorporates economic evaluation to explore the cost-effectiveness of the intervention.

### Conclusions

This feasibility pilot trial was designed and conducted to test the essential aspects of the research design and acceptability of the intervention to examine the potential for conducting a future fully powered RCT. Despite evidence suggesting the acceptability of PUfP, the findings suggest that recruitment modifications are needed to enhance the feasibility of collecting follow-up data before scaling up to a full RCT. Possible implications for practice and policy were discussed alongside recommendations for future research. One important recommendation is that the future RCT may benefit from incorporating a mechanism to explore the cost-effectiveness of implementing PUfP. Furthermore, in recognition of the age and capacity of young people and the promotion of standards of care to empower young service users, considerations for refining PUfP to interact with other versions of Power Up may be valuable.

## Appendix

Multimedia Appendix 1 Summary of findings against 8 predetermined Go-No-Go criteria for feasibility research.

Multimedia Appendix 2 Characteristics of parents participating in interviews and focus group discussions.

Multimedia Appendix 3 Characteristics of HCPs participating in interviews and focus group discussions.

Multimedia Appendix 4 Demographic characteristics of parent participants in stage 2 of the feasibility trial.

Multimedia Appendix 5 Summary of outcome data.

Multimedia Appendix 6 CONSORT-EHEALTH checklist (V.1.6.1).

## Abbreviations

CPS: Control Preference Scale
CYPMH: children’s and young people’s mental health
DCS: Decisional Conflict Scale
ESQ: Experience of Service Questionnaire
FGD: focus group discussion
NHS: National Health Service
PPI: patient and public involvement
PSSUQ: Poststudy System Usability Questionnaire
PUfP: Power Up for Parents
RCT: randomized controlled trial
REC: research ethics committee
RQ: research question
SDM: shared decision-making
TAM: Technology Acceptance Model
