# Supplementary material for: Feasibility and Acceptability of a Digital Intervention to Support Shared Decision-making in Children’s and Young People’s Mental Health: Mixed Methods Pilot Randomized Controlled Trial
Source: JMIR Form Res. 2021 Mar 2;5(3):e25235. doi: 10.2196/25235 (PMC7967225; doi:10.2196/25235)
Supplement: Multimedia Appendix 1 [file formative_v5i3e25235_app1.docx]

Multimedia Appendix 1 Summary of findings against 8 predetermined 8 Go-No-Go criteria for feasibility research

| Criteria Description | Findings | Assessment of readiness (score) | Score achieved |
| --- | --- | --- | --- |
| Parents reporting PUfP as acceptable in interviews and outcome measures | 78.57 of parents participating in the interviews (11/14) expressed satisfaction with the intervention and provided an average score of 3.15 on the overall PSSUQ measure. | 0-20% of parents report that PUfP is acceptable (0) |  |
|  |  | 21-49% of parents report PUfP is acceptable (1) |  |
|  |  | 50-100% of parents report PUfP is acceptable (2) | 2 |
| Parents reporting PUfP as useful in interviews and outcome measures | 92.86% of the parents participating in the interviews (13/14) provided feedback highlighting that the intervention was useful and provided an average score of 3.13 on the PSSUQ subscale for usefulness. | 0-20% of parents report that PUfP is useful (0) |  |
|  |  | 21-49% of parents report PUfP is useful (1) |  |
|  |  | 50-100% of parents report PUfP is useful (2) | 2 |
| HCPs reporting PUfP as acceptable in interviews | 78.95% of HCPs in the sample (15/19) provided favourable feedback on the intervention. | 0-20% of HCPs report that PUfP is acceptable (0) |  |
|  |  | 21-49% of HCPs report PUfP is acceptable (1) |  |
|  |  | 50-100% of HCPs report PUfP is acceptable (2) | 2 |
| HCPs reporting PUfP as useful in interviews | 84.21% clinicians (16/19) expressed that the intervention was useful. | 0-20% of HCPs report that PUfP is useful (0) |  |
|  |  | 21-59% of HCPs report PUfP is useful (1) |  |
|  |  | 50-100% of HCPs report PUfP is useful (2) | 2 |
| Ability to recruit and retain sites | 66.67% of CYPMHS sites (12/18) were recruited into the study. One site withdrew after 3 months, and the remaining 11 (61.11%) were able to recruit participants for stage 1 and/or stage 2 of the study. | 0-19% of sites recruited and retained (0) |  |
|  |  | 20-49% of sites recruited and retained (1) |  |
|  |  | 50-100% of sites recruited and retained (2) | 2 |
| Site’s ability to recruit and complete baseline measures | 50% of the sites (6/12) were able to recruit participants and obtain baseline data for stage 2 of the study. | 0-19% of sites recruited and retained (0) |  |
|  |  | 20-49% of sites recruited and retained (1) | 2 |
|  |  | 50-100% of sites recruited and retained (2) |  |
| Rates of access and usage of PUfP | 117 users accessed PUfP and 72 (61.54%) registered an account. It is estimated that the majority of the intervention arm participants (n=~30) accessed the intervention contributing to these statistics. | 0-19% of parents access and use PUfP (0) |  |
|  |  | 20-49% of parents access and use PUfP (1) | 2 |
|  |  | 50-100% of parents access and use PUfP (2) |  |
| Ability to retain participants and complete follow-up measures | Of the 42 parents in the study, 16 (38.1%) completed follow-up measures (i.e. 12 from NHS and 4 from the Community sample). | 0-19% of participants retained and completed follow-up measures (0) | 1 |
|  |  | 20-49% of participants retained and completed follow-up measures (1) |  |
|  |  | 50-100% of participants retained and completed follow-up measures (2) |  |
|  |  |  |  |
|  |  |  |  |
|  | Aggregate meaning | |  |
|  |  |  | Total Score (15) |
|  | Ready to proceed to full RCT | The criteria have been attained | *12-16 |
|  |  |  |  |
|  |  |  |  |
|  | Ready with some action to be taken | The criteria have not been attained but an existing plan can | *6-11 |
|  |  | bring attainment of the criteria or allow the RCT to proceed |  |
|  |  | absence of attainment |  |
|  |  |  |  |
|  | Not ready | The criteria have not been attained and there is no existing plan than can bring attainment of the criteria | *0-5 |
|  |  |  |  |
