# Supplementary material for: Feasibility and Acceptability of a Digital Intervention to Support Shared Decision-making in Children’s and Young People’s Mental Health: Mixed Methods Pilot Randomized Controlled Trial
Source: JMIR Form Res. 2021 Mar 2;5(3):e25235. doi: 10.2196/25235 (PMC7967225; doi:10.2196/25235)
Supplement: Multimedia Appendix 2 [file formative_v5i3e25235_app2.docx]

Multimedia Appendix 2 Characteristics of parents participating in interviews and focus group discussions

| Variable | Interviews (n=14) | FGDs (n=2) | Total sample (n=24) |
| --- | --- | --- | --- |
| Parent’s age  Mean (SD)  Range | 45.93 (6.12)  36-53 | 43.4 (7.65)  31-54 | 44.88 (6.76)  31-54 |
| Relationship to child n(%)  Mother  Father | 14 (100)  0 (0) | 8 (80)  2 (20) | 22 (91.67)  2 (8.33) |
| Ethnicity n(%)  White  Other | 14 (100)  0 (0) | 9 (90)  1 (10) | 23 (95.83)  1 (4.17) |
| CYP’s age  Mean (SD)  Range | 14.36 (3.61)  8-22 | 13.2 (0.63)  13-14 | 13.88 (2.8)  8-22 |
| CYP’s gender n(%)  Male  Female  Other | 5 (35.71)  9 (64.29)  0 (0) | 2 (20)  7 (70)  1 (10) | 7 (29.17)  16 (66.67)  1 (4.17) |
| ^a^CYP’s clinical characteristics n(%)  ^b^ADHD  Anxiety  ^c^ASD  Depression  ^d^PTSD  Comorbidities*  Undiagnosed | 1 (7.14)  0 (0)  1 (7.14)  2 (14.29)  1 (7.14)  8 (57.14)  1 (7.14) | 0 (0)  4 (40)  0 (0)  0 (0)  0 (0)  0 (0)  6 (60) | 1 (4.17)  4 (16.67)  1 (4.17)  2 (8.33)  1 (4.17)  8 (33.33)  7 (29.17) |

*Comorbidities included a subset of ADHD, Anxiety, ASD, Depression, self-harm, suicide attempt, psychosis and Asperger’s Syndrome

^a^Children and young people; ^b^Attention Deficit and Hyperactivity Disorders; ^c^Autism Spectrum Disorders; ^d^Post-Traumatic Stress Disorders; SD= Standard deviation
