# Supplementary material for: Feasibility and Acceptability of a Digital Intervention to Support Shared Decision-making in Children’s and Young People’s Mental Health: Mixed Methods Pilot Randomized Controlled Trial
Source: JMIR Form Res. 2021 Mar 2;5(3):e25235. doi: 10.2196/25235 (PMC7967225; doi:10.2196/25235)
Supplement: Multimedia Appendix 3 [file formative_v5i3e25235_app3.docx]

Multimedia Appendix 3 Characteristics of HCPs participating in interviews and focus group discussions

| Variable | Interviews (n=19) | ^a^FGDs* (n=2) | Total sample (n=31) |
| --- | --- | --- | --- |
| Occupation n(%)  Psychiatrist  Psychologist/Psychotherapist  Nurse  Other* | 4 (21.05)  2 (10.53)  2 (10.53)  11 (57.89) | 1 (8.33)  5 (41.67)  4 (33.33)  2 (16.67) | 6 (19.35)  9 (29.03)  6 (19.35)  10 (32.26) |
| Clinical expertise n(%)  Eating disorders  General* | 2 (10.53)  17 (89.47) | 0 (0)  12 (100) | 2 (6.45)  29 (93.55) |
| Experience in CAMHS (years)  Mean (SD)  Range | 6.36 (5.87)  0.58-20 | 9.40 (6.62)  2.25-20 | 7.54 (6.24)  0.25-22 |

*Other represents Registrar, Occupational Therapist, Social Worker, Support Worker and Team Manager.

*Working in general children and youth MH settings which includes, but not limited to, behavioural, attention deficit and autism spectrum disorders.

^a^Focus group discussions
