# Supplementary material for: Feasibility and Acceptability of a Digital Intervention to Support Shared Decision-making in Children’s and Young People’s Mental Health: Mixed Methods Pilot Randomized Controlled Trial
Source: JMIR Form Res. 2021 Mar 2;5(3):e25235. doi: 10.2196/25235 (PMC7967225; doi:10.2196/25235)
Supplement: Multimedia Appendix 4 [file formative_v5i3e25235_app4.docx]

Multimedia Appendix 4 Demographic characteristics of parent participants in stage 2 of the feasibility trial

| Variable | Clinic n=30  Mean (SD) or n (%) | Community n=12  Mean (SD) or n (%) | Total N=42  Mean (SD) or n (%) |
| --- | --- | --- | --- |
| Relationship to child |  |  |  |
| Mother | 24 (80%) | 12 (100%) | 36 (85.71%) |
| Father | 4 (13.33%) | 0 | 4 (9.52%) |
| Other | 2 (6.67%) | 0 | 2 (4.76%) |
| Parent’s age in years | 46.10 (6.85) | 45.67 (5.66) | 45.98 (6.45) |
| Ethnicity |  |  |  |
| White | 28 (93.33%) | 11(91.67%) | 39 (92.86%) |
| Black | 1 (3.33%) | 0 | 1 (2.38%) |
| Asian | 1 (3.33%) | 0 | 1 (2.38%) |
| Mixed | 0 | 1 (8.33%) | 1 (2.38%) |
| English as 1^st^ language: Yes | 28 (93.33%) | 11(96.67%) | 39 (92.86%) |
| Child’s age in years | 14.6 (2.16) | 13.58 (1.98) | 14.31 (2.14) |
| Child’s gender  Male  Female  Other | 7 (23.33%)  22(73.33%)  1 (3.33%) | 4 (33.33%)  8 (66.67%)  0 | 11 (26.19%)  30 (71.43%)  1 (2.38%) |
