# Supplementary material for: Feasibility and Acceptability of a Digital Intervention to Support Shared Decision-making in Children’s and Young People’s Mental Health: Mixed Methods Pilot Randomized Controlled Trial
Source: JMIR Form Res. 2021 Mar 2;5(3):e25235. doi: 10.2196/25235 (PMC7967225; doi:10.2196/25235)
Supplement: Multimedia Appendix 5 [file formative_v5i3e25235_app5.docx]

Multimedia Appendix 5 Summary of outcome data

| Outcomes measure | All participant  Mean(SD) or n(%) | | IG1  Mean(SD) or n(%) | | IG2  Mean(SD) or n(%) | | Control  Mean(SD) or n(%) | |
| --- | --- | --- | --- | --- | --- | --- | --- | --- |
|  | Baseline N=42 | Follow-up n=16 | Baseline n=11 | Follow-up n=1 | Baseline n=19 | Follow-up n=4 | Baseline n=12 | Follow-up n=11 |
| **^a^CPS_P**  ^b^HCP-lead  ^c^SDM  Parent-lead | 5 (11.9%)  26 (61.9%)  11 (26.2%) | 2 (12.5%)  10 (62.5%)  4 (25%) | 2(18.18%)  8 (72.72%)  1(9.09%) | 0  1  0 | 1(5.26%)  10 (52.63%)  8 (26.32%) | 0  2 (50%)  2 (50%) | 2 (16.67%)  8 (66.67%)  2 (16.67%) | 2(18.18%)  7 (63.64%)  2 (18.18%) |
| **Observer**  HCP-only  HCP-lead  SDM  Parent-lead  Missing data | 1 (2.38%)  6 (14.29%)  5 (11.9%)  4(9.52%)  16 (38.1%) | 1(6.25%)  1(6.25%)  4(25%)  1(6.25%)  7(43.75%) | 0  3(27.27%)  3(27.27%)  1 (6.25%)  4(36.36%) | 0  0  3  1 | 0  0  0  3(15.79%) | 0  0  0  0 | 1(8.33%)  3(25%)  2(16.67%)  0  6(50%) | 1 (9.09)  1 (9.09)  1 (9.09)  0  8 (72.72) |
| ^d^PSDM-Q-9 | 26.54 (0.98) | 28.81(10.48) | 28.45(9.23) | 33 | 24.28(12.62) | 26.25(13.28) | 28.17(9.62) | 29.36(10.36) |
| **^e^STAI-AD** |  |  |  |  |  |  |  |  |
| STATE  TRAIT | 40.85(14.12)  45.9(13.4) | 44.25(16.10)  48.88(11.63) | 43.18(9.88)  47.55(9.85) | 58  60 | 38.37(17.67)  43.26(15.97) | 46.5(22.35)  53.5(17.17) | 42.67(12.59)  48.58(12.06) | 42.18(14.61)  46.18(14.61) |
| **Total ^f^DCS**  Uncertainty  Informed  Values  Supported  Effective | 35.44(17.85)  41.67(25.69)  36.59(21.48)  27.5 (19.81)  40.24(26.41)  32.77(18.74) | 38.18(19.22)  43.75(21.62)  38.54(20.15)  31.25(23.27)  41.67(24.72)  36.45(18.73) | 35(21.59)  40.83(28.72)  29.17(19.35)  29.17(23.98)  43.33(28.27)  33.13(25.86) | 31.25  58.33  25  25  25  25 | 39.14(18.6)  48.68(25.8)  43.42(24.15)  27.78 (21)  46.93(27.68)  32.57(19.61) | 45.31(26.03)  50(29.66)  50(24.53)  29.17(33.68)  56.25(36.24)  45.83(25.91 | 29.95(12.46)  31.25(20.76)  31.94(16.22)  25.69(15.26)  27.08(18.84)  32.81(10.02) | 36.22(17.91)  40.15(19.66)  35.61(18.67)  32.57(21.56)  37.88(19.85)  34.09(16.44) |
| **^g^Satisfaction** |  |  |  |  |  |  |  |  |
| Care  Service  SDM | 20.62(5.74)  27.05(27.05)  9.1(2.45) | 19.63(6.93)  26.79(7.64)  8.56(3.1) | 23.27(3.85)  30.18(4.21)  10.18(2.14) | 26  33  12 | 20.11(6.01)  26.47(7.03)  8.87(2.51) | 15.5(8.06)  21.75(8.81)  6.5(3.79) | 19(6.32)  25.08(6.89)  8.33(2.46) | 20.55(6.38)  27.18(7.12)  9(2.65) |
| **^h^PSSUQ**  Usefulness  Information  Interface |  | 3.15 (0.63)  3.13 (0.66)  3.0 (0.54)  3.42 (1.17) |  |  |  |  |  |  |

Note: IG = Intervention Group; SD= Standard Deviation; ^a^Control Preference Scale for Paediatrics; ^b^Healthcare Professional; ^c^Shared Decision-Making; ^d^9-item Paediatric Shared Decision-Making Questionnaire; ^e^Spielberger State Anxiety Inventory Form for Adults; ^f^Decisional Conflict Scale; ^g^Experience of Service Questionnaire; ^h^Post-Study Usability Questionnaire
